# Supplementary material for: Longitudinal healthcare use after pediatric brain injury: A population-based birth cohort study
Source: PLoS One. 2025 Feb 24;20(2):e0316165. doi: 10.1371/journal.pone.0316165 (PMC11849829; doi:10.1371/journal.pone.0316165)
Supplement: S1 Table — (PDF) [file pone.0316165.s001.pdf]

Diagnosis Codes	
**OHIP**	803: Fracture, fracture-dislocation, skull<br><br>850: Concussion<br><br>854: Injury, head
**ICD10**	S02.0: Fracture of vault of skull<br><br>S02.1: Fracture of base of skull<br><br>S02.3: Fracture of orbital floor<br><br>S02.7: Multiple fractures involving skull and facial bones<br><br>S02.8: Fracture of other skull and facial bones<br><br>S02.9: Fracture of skull and facial bones, part unspecified<br><br>S04.0: Injury of optic nerve and pathways<br><br>S06: Intracranial injury<br><br>S07.1: Crushing injury of skull<br><br>T02.0: Fracture involving head with neck<br><br>T06.0: Injuries of brain and cranial nerves with injuries of nerves and spinal cord at neck level
