# Supplementary figures and images for: Longitudinal healthcare use after pediatric brain injury: A population-based birth cohort study
Source: PLoS One. 2025 Feb 24;20(2):e0316165. doi: 10.1371/journal.pone.0316165 (PMC11849829; doi:10.1371/journal.pone.0316165)

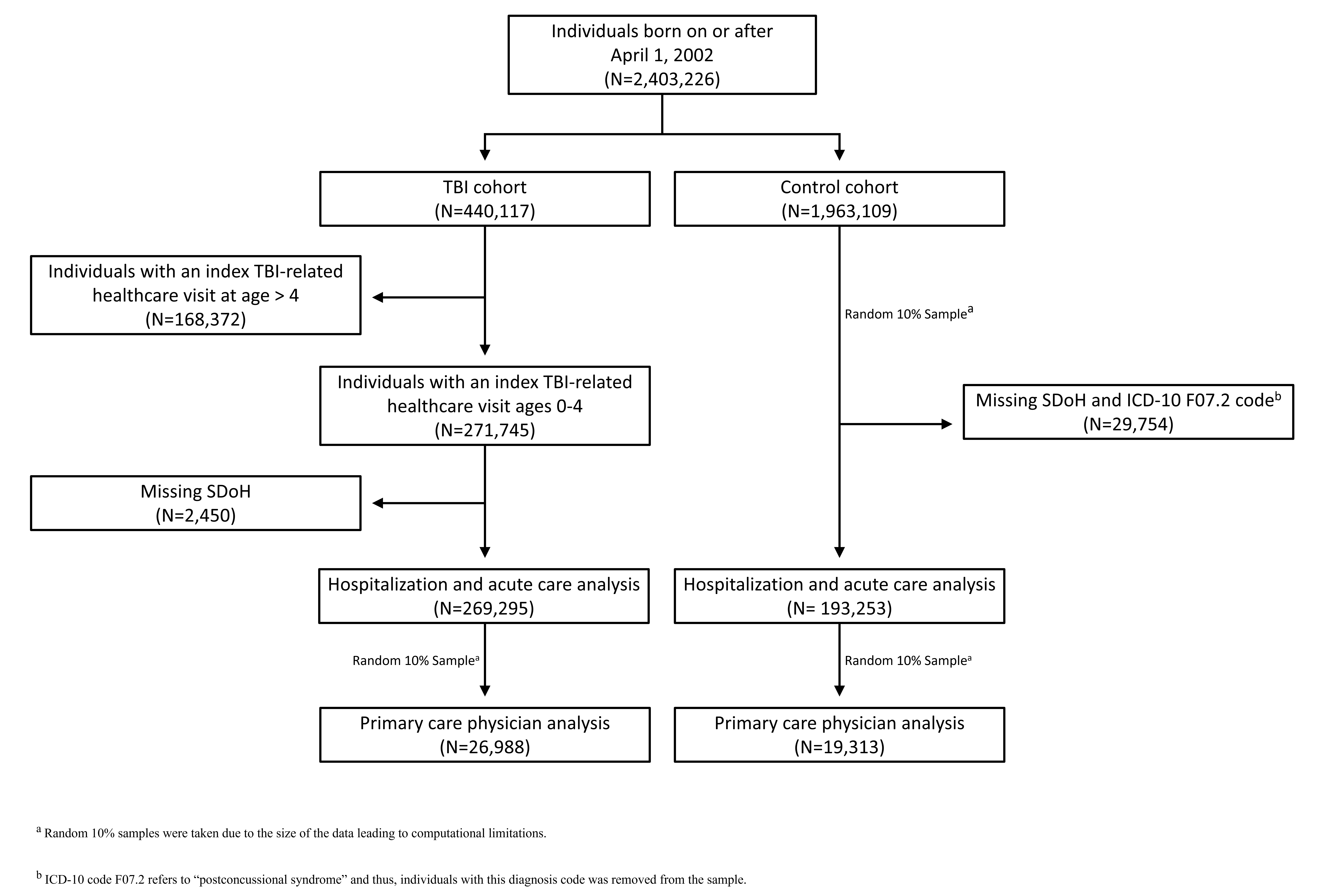

Supplement: S1 Fig — (TIF) [file pone.0316165.s002.tif]
