# Supplementary material for: Longitudinal healthcare use after pediatric brain injury: A population-based birth cohort study
Source: PLoS One. 2025 Feb 24;20(2):e0316165. doi: 10.1371/journal.pone.0316165 (PMC11849829; doi:10.1371/journal.pone.0316165)
Supplement: S1 Text — (PDF) [file pone.0316165.s007.pdf]

This study had computation limitations with regards to processing power and time. Due to the large number of healthcare records (often in the millions), we reduced the sample size by taking a random sample of the TBI and control cohort, where required. Specifically, for analyses on the rates of emergency department and hospitalizations, all patients who experienced an index TBI-related healthcare visit between the ages of 0 and 4 years, inclusive, were included (n=269,295). However, a random 10% of patients in the control cohort was taken to reduce the number of controls from n=1,963,109 to n=193,253. Similarly, primary care physician visits occurred much more frequently compared to other healthcare settings. As such, a further random 10% sample for both the TBI cohort and the control was taken to reduce the same to n=26,988 for the TBI cohort and n=19,313 for control.
